# *Helicobacter pylori* and Compositional Patterns of Digestive Tract Microbiome in Children: A Literature Review

**DOI:** 10.3390/nu17162711

**Published:** 2025-08-21

**Authors:** Ancuta Lupu, Anca Adam-Raileanu, Laura Iulia Bozomitu, Nicoleta Gimiga, Lorenza Forna, Carmen Rodica Anton, Maria Oana Sasaran, Alin Horatiu Nedelcu, Dragos Catalin Ghica, Emil Anton, Ionela Daniela Morariu, Silvia Fotea, Omer Faruk Beser, Vasile Valeriu Lupu

**Affiliations:** 1 Faculty of Medicine, “Grigore T. Popa” University of Medicine and Pharmacy, 700115 Iasi, Romania; anca_ign@yahoo.com (A.L.); chiti_nico@yahoo.com (N.G.); lorenza.donea@yahoo.ro (L.F.); carmen.anton@umfiasi.ro (C.R.A.); alin_nedelcu@yahoo.com (A.H.N.); dragos.ghica@yahoo.ro (D.C.G.); emil.anton@yahoo.com (E.A.); valeriulupu@yahoo.com (V.V.L.); 2 Faculty of Medicine, “George Emil Palade” University of Medicine, Pharmacy, Science and Technology, 540142 Targu Mures, Romania; oanam93@yahoo.com; 3 Faculty of Pharmacy, “Grigore T. Popa” University of Medicine and Pharmacy, 700115 Iasi, Romania; ionela.morariu@umfiasi.ro; 4 Faculty of Medicine and Pharmacy, “Dunarea de Jos” University of Galati, 800008 Galati, Romania; silvia_ghimpu@yahoo.com; 5 Department of Pediatric Gastroenterology, Hepatology & Nutrition, Cerrahpasa Medical Faculty, Istanbul University Cerrahpasa, Istanbul 34776, Turkey; ofbeser@gmail.com

**Keywords:** *Helicobacter pylori*, digestive tract, microbiota, children

## Abstract

*Helicobacter pylori* (*H. pylori)* represents a major healthcare problem, colonizing more than half of the population worldwide. Usually acquired during childhood, it has a significant impact on human health. After forty years of extensive research, there are aspects of the complex *H. pylori*–human organism interplay that require further investigation. A comprehensive review was conducted after an extensive literature search in the PubMed/Medline, Web of Science, and EMBASE databases concerning *H. pylori* and human microbiota reports. Although the exact nature of *H. pylori*’s relation with the human microbiome remains elusive, its presence as well as its eradication treatment are associated with the alteration of bacterial communities’ composition not only in the gastric microenvironment but also in all digestive tract levels, with particular changes in both children and adults. Understanding microbiota composition is a step towards personalized medicine. Although the current literature on pediatric patients related to this topic is scarce, the available positive results reported in adult studies encourage pediatric research on microbiota manipulation, promising beneficial outcomes.

## 1. Introduction

Currently, novel techniques such as targeted sequencing of the 16S RNA gene, whole-metagenome shotgun sequencing, or other metagenomic technologies are allowing significant advances in human microbiome study. An impressive body of literature is focused on characterizing the composition, dynamics, and activity of the human microbiome and microbiota-related inter-bacterial interactions [1,2].

### 1.1. Helicobacter pylori—A Longstanding Medical Challenge

If human microbiome study has brought novelty in medical research, opening up a new chapter in human biological study, *Helicobacter pylori* (*H. pylori*) stands in opposition. It represents one of the most debated subjects of scientific research, with a history of over 40 years of medical study. The *H. pylori*–human being relationship has a complex nature that has evolved over thousands of years, with *H. pylori* infecting almost half of the world’s population [3,4,5].

Although most research conducted in the past few decades has highlighted a downward trend in *H. pylori* prevalence throughout global geographic areas, *H. pylori* infection can still be considered a significant clinical and public health problem [6,7].

### 1.2. Transmission Pathways of Helicobacter pylori

Vertical and horizontal transmission are the two primary types into which person-to-person transmission may be classified. Whereas horizontal transmission occurs through interaction with people outside the familial environment or through environmental contamination, vertical transmission occurs when an illness spreads from ascendant to descendant within the same family members [8,9]. As a matter of fact, there are several studies concerned with intrafamilial infection exposure [10,11,12]. In addition to sharing a genetic predisposition to *H. pylori* infection, it is hypothesized that person-to-person transmission occurs within the same family, possibly because of close interpersonal contacts, exposure to a common source of infection, and social and economic status. While infected mothers seem to represent the main source of infection in countries with a high economic status, in countries with low socioeconomic conditions, the dissemination route seems to be facilitated by sibling transfer and external acquisition [13,14].

### 1.3. Pathogenesis and Genetic Adaptability of Helicobacter pylori

*H. pylori* colonization and pathogenicity involve several steps. First of all, its survival under acidic environment offered by the stomach is assured by its urease secretion, which converts urea into ammonia, neutralizing stomach acid; in terms of adhesion, the bacteria are able to adhere to the gastric epithelium using specific surface adhesins, allowing them to resist being removed by gastric acid and mucus; furthermore, *H. pylori* triggers an inflammatory response through the release of cytokines and other inflammatory mediators, leading to chronic inflammation (chronic gastritis) [15]. The bacteria also produce enzymes and toxins (such as CagA) that can damage gastric epithelial cells, disrupt the tight junctions between cells, and lead to the loss of protective mucosal layers. This damage can also cause ulcers and, over time, increase the risk of gastric cancer. Nevertheless, *H. pylori* is known for its remarkable genetic adaptability, which is primarily attributed to its high recombination rate. This genetic flexibility allows the bacterium to adapt to various environments, escape immune responses, and potentially develop resistance to antibiotics [16]. *H. pylori* has a unique life cycle and replicative strategy that leads to a high genetic recombination rate, which facilitates the rapid exchange of genetic material among strains [17,18]. Furthermore, the genome of *H. pylori* is characterized by significant plasticity, with variations in gene content that can be traced back to horizontal gene transfer and recombination events. In addition, there is proof that *H. pylori*’s genetic adaptability allows it to acquire resistance to antibiotics through the incorporation of resistance genes from other bacteria, contributing to the emergence of antibiotic-resistant strains of *H. pylori* [19,20].

### 1.4. Challenges and Side Effects of Antibiotic Therapy

Whether via the oral–fecal route, oral–oral route, or environmental exposure, once the presence of *H. pylori* is confirmed, the same debate on whether to treat pediatric *H. pylori* infection will start. In current practice, it is no longer possible to support the triple or sequential therapy recommendations made by previous guidelines for naïve children and adolescents [6,7]. Rather, therapy should be based on data concerning antibiotic resistance profiles and customized accordingly utilizing sufficiently high dosages and treatment periods of 10 to 14 days to achieve an initial eradication success rate of 90% or higher. Only patients with infections caused by strains sensitive to clarithromycin should be part of the regimen including this antibiotic. High-dose triple therapy with PPIs (proton pomp inhibitors), amoxicillin, and metronidazole for 14 days is advised as the first-line treatment when antibiotic susceptibility profiles are unknown. PPIs are drugs that irreversibly inhibit the H^+^/K^+^ ATPase enzyme system at the surface of the gastric parietal cells, effectively suppressing gastric acid secretion [21]. In nations where it is authorized for use in children, bismuth-based quadruple therapy may also be taken into consideration as a first-line treatment [22]. Antibiotic treatment is a widely used approach to eradicate *H. pylori* infection in pediatric patients and prevent its associated complications. While antibiotics can be effective in treating *H. pylori*, they may also have several side effects, including gastrointestinal disturbances, alteration of gut microbiota, impact nutrient absorption, allergic reactions, and antibiotic resistance development [23]. According to Bontems et al., among pediatric patients undergoing *H. pylori* eradication therapy, 20% had stomach discomfort (sequential 24% vs. triple therapy 17%), 14% experienced diarrhea (12% vs. 16%), 6% experienced nausea (8% vs. 5%), and 2% experienced vomiting (4% vs. 0%) [24].

### 1.5. Alternative and Complementary Therapies

To diminish the classic eradication scheme associated with an adverse reaction, several naturally derived compounds have been proposed for treating *H. pylori* infection. Extracts from Terminalia chebula Retz and Syzygium aromaticum are only a few of the *H. pylori* alternative treatment options that have been successful in adult studies [25,26,27]. However, further research is required to prove their efficacy and safety in both adult and pediatric populations. Probiotics have been also proposed to increase adherence and lessen negative effects in eradication regimens [28,29,30,31]. It is a well-known fact that prebiotics and probiotics are important contributors to the human organism’s general state of health and nutrition. Probiotics are “live microorganisms that, when administered in adequate amounts, confer a health benefit on the host”, according to the International Scientific Association for Probiotics and Prebiotics, while prebiotics can be considered non-digestible compounds that promote the growth and activity of beneficial microbial species, including bacteria and fungi [32].

According to current research in both adult and pediatric patients, certain probiotic strains, such *Lactobacillus* and *Bifidobacterium*, could inhibit *H. pylori*’s growth. Furthermore, they may be used in association with antibiotics to decrease the bacterial load, alleviate symptoms, and improve the efficacy of *H. pylori* eradication therapy [33]. On the other hand, by providing nourishment for beneficial bacteria, prebiotics can help enhance the overall diversity of the gut microbiota, which may indirectly limit the overgrowth of harmful bacteria like *H. pylori*. Both probiotics and prebiotics have the capacity of positively influencing children’s intestinal microbiota and may play a role in managing *H. pylori* infections. However, further research in pediatric patients is required [34].

### 1.6. Microbiota and Personalized Medicine—Material and Methods

Given the decades of intensive research, it would be easy to believe that the medical scientific community would not obtain any new insights from *H. pylori* study. However, there are developments in modern technology which may be applied. Furthermore, the available literature offers a comprehensive understanding of the numerous aspects of *H. pylori* infection and its important systemic implications in both children and adults [35,36], with an impressive number of studies on the adult population, but their results cannot be extended to pediatric patients as children seem to display distinct features in their microbial pattern [37,38,39,40].

A comprehensive review was conducted after an extensive literature search in the PubMed/Medline, Web of Science, and EMBASE databases concerning *H. pylori* and human microbiota reports. The search was limited to English-language articles published between January 1995 and June 2025. The search terms used were as follows: (“*Helicobacter pylori*” OR “*H. pylori*”) AND (“microbiome” OR “microbiota” OR “gut flora” OR “intestinal microbiota”) AND (“child” OR “children” OR “pediatric” OR “infant” OR “adolescent”) AND (“digestive tract” OR “gastrointestinal” OR “oral microbiome” OR “gut microbiome”). Boolean operators (AND, OR) were used to combine and refine search terms appropriately. We included articles assessing microbiome composition in relation to *H. pylori* infection, studies using sequencing techniques (e.g., 16S rRNA sequencing, metagenomics), original research articles, clinical studies, and systematic reviews. Articles not addressing microbiome composition or diversity, case reports, editorials, and opinion papers were excluded from analysis. We must report the lack of information regarding the relationship between *H. pylori* and children’s oral microbiota, as most available studies are focused on the adult population.

### 1.7. Aim of Current Study and Future Directions

Nevertheless, we share the belief that the relation of *H. pylori*–human microbiota has a major influence on one’s state of health and disease. Characterizing the microbial compositional shifts determined by *H. pylori*’s presence or its eradication therapy represents only a small step towards the goal of a personalized medicine that will offer efficient management of this worldwide health problem. This narrative review provides insightful information on the current understanding of *H. pylori* infection through a critical synthesis of recent research findings.

While current evidence underscores the importance of gut microbiota in gastrointestinal health, several critical gaps remain. First, most probiotic and microbiota-modulating trials have been conducted in adults, leaving an urgent need for well-designed, pediatric-specific randomized controlled trials (RCTs) that can inform age-appropriate recommendations. Second, oral–gut microbial interactions remain underexplored; noninvasive oral screening for pathogens such as *H. pylori* could provide an accessible tool for early detection and risk stratification, especially in children and adolescents. Finally, the development of age-tailored strategies for microbiota modulation—whether through diet, probiotics, or next-generation therapeutics—may offer more precise and durable benefits than current one-size-fits-all approaches. Addressing these gaps will be essential to move from observational associations toward targeted interventions that can improve prevention, diagnosis, and long-term outcomes in both pediatric and adult populations, limiting *H. pylori*’s negative influence on patients’ lives and reducing associate healthcare costs.

## 2. Microbiome Maturation and Its Role in *Helicobacter pylori* Colonization and Treatment Response

**Figure 1:**
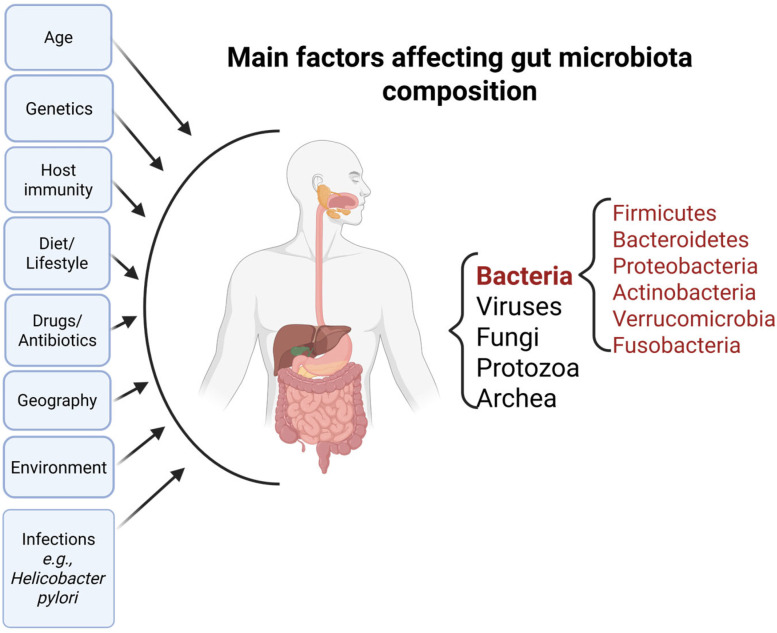
Human digestive tract associated microbiota and main factors affecting its composition.

The human microbiome is described as the genetic content of microorganisms (microbiota) that live at a specific location within the body of each individual [41]. It contains bacteria, viruses, protozoans, fungi, and archaea with more than 10^13^ microbial cells [42]. Numerous projects such as the Human Microbiome Project (HMP) [43] and MetaHIT [44] focused their attention and resources on characterizing a healthy human microbiome composition, with the aim of achieving a better understanding of the microbial population living within our organisms and their influence on our state of health and disease. With a dynamic content, the diversity and richness of the microbiome is shaped by numerous factors, including age, sex, delivery type, feeding practices, lifestyle, pathogens, different external environmental factors as well as internal setting parameters (pH, humidity)—as illustrated in Figure 1 [45,46,47]. The GI tract hosts the highest number of microbes, due to its nutrient bioavailability. Represented by six major phyla such as *Firmicutes*, *Bacteroidetes*, *Proteobacteria*, *Actinobacteria*, *Verrucomicrobia*, and *Fusobacteria*, its composition varies with each anatomical component of the GI tract, sharing a common core [48,49].

The composition and function of the human microbiome undergo profound changes throughout early life, from infancy to adolescence. This dynamic process of microbial maturation is fundamental in the gastrointestinal tract, where the establishment of a balanced and diverse microbiota plays a pivotal role in immune system development, mucosal barrier function, and pathogen defense. In the context of *H. pylori* infection, these age-related microbial transitions may significantly influence both colonization success and therapeutic response in pediatric patients [1,2].

### 2.1. Microbial Development from Infancy to Adolescence

Recent research has revealed that the development of the microbiome begins during prenatal life and increases its richness and diversity afterwards, during childhood and adolescence. With different functional and compositional patterns in the gut microbiome between pediatric and adult populations, children’s microbiome is less stable and diverse.

During infancy, gut microbiota is characterized by low diversity and instability, heavily influenced by mode of delivery, breastfeeding, antibiotic exposure, and early diet. The microbial composition gradually transitions from being dominated by facultative anaerobes (e.g., Enterobacteriaceae) to a more complex ecosystem rich in Bacteroidetes and Firmicutes by 2–3 years of age [50]. This process continues into later childhood and adolescence, during which the microbiome matures toward an adult-like configuration, with increasing taxonomic and functional stability.

In parallel, the oral and gastric microbiomes also mature, influenced by dietary diversification, dental eruption, and hormonal changes. These changes affect microbial niches and competition, potentially influencing *H. pylori*’s ability to colonize or persist [51].

### 2.2. Implications for H. pylori Colonization

The immature microbiome in young children may provide reduced colonization resistance, allowing *H. pylori* to establish infection more easily due to lower microbial competition. Furthermore, it might exhibit distinct immune priming effects, potentially altering the host’s inflammatory response to *H. pylori*. Lastly, an immature microbiome could be more susceptible to disruption by external factors such as antibiotics, which can unintentionally favor *H. pylori* persistence or reinfection [2].

Conversely, as the microbiome matures, a more stable and competitive microbial environment might limit the window of opportunity for primary colonization. Additionally, it could influence strain-specific outcomes depending on co-colonizing species.

### 2.3. Treatment Response and Microbial Maturity

Age-related differences in microbial composition and function may also influence treatment efficacy. For instance, younger children may experience greater disruption of the gut microbiota following antibiotic therapy, potentially leading to delayed microbial recovery or increased side effects. The presence of specific beneficial taxa in older children (e.g., Lactobacillus, Bifidobacterium) may support improved eradication outcomes when used alongside probiotic supplementation. Pharmacokinetics and acid secretion patterns, both influenced by age, can also impact the bioavailability of PPIs and antibiotics, further modulating therapy success [9,42].

Despite these insights, current treatment guidelines rarely stratify recommendations based on microbiome maturity or age-specific microbial profiles [52,53,54]. Longitudinal studies are needed to determine the optimal age for intervention based on microbiome resilience. Identifying microbial biomarkers predictive of colonization susceptibility or treatment failure represents another current research gap that requires additional investigation.

## 3. *Helicobacter pylori* and Microbiome Study

*H. pylori* is a Gram-negative bacteria found in the gastric mucosa of both children and adults. Once considered to be a sterile organ because of its severe environment, it is now well acknowledged that the stomach has a distinct microbiome, even if the bacterial count is just around 10^2^–10^4^ CFU/mL [10]. Traditional culture-based techniques used initially in gastric microbiota study were inaccurate and biased since 80% of the gastric bacteria are non-cultivable [55]. It was only in 1983 that *H. pylori* was cultured from human gastric mucosa by Marshall and Warren, enabling physicians and researchers all over the world to understand the infectious etiology of some of the gastric diseases [56].

### 3.1. Modern Diagnostic Approaches for H. pylori Infection

Currently, the diagnosis of *H. pylori* employs various methods, including stool antigen tests, real-time PCR, and endoscopic biopsies [26]. While these tests are effective for detecting *H. pylori*, integrating an understanding of the microbiome can provide several added advantages. By examining the entire gut microbiome composition rather than focusing solely on *H. pylori*, healthcare professionals can gain insights into the overall microbial balance. Analyzing the broader microbiota can reveal additional dysbiosis, which may impact the severity of *H. pylori*-related conditions and overall gastrointestinal health. Understanding an individual’s microbiome composition can further enhance tailored treatment strategies, increasing eradication rates. The microbiome can influence antibiotic resistance. Studying microbial communities may help predict which antibiotics will be most effective in eradicating *H. pylori* based on the individual’s microbial composition, thereby optimizing treatment protocols. In addition, as for monitoring treatment efficacy, assessing changes in the gut microbiome following *H. pylori* treatment can provide valuable feedback on the effectiveness of the therapy. Analyzing shifts in microbial populations can indicate whether the treatment is restoring a healthier gut environment [42,57].

During the last decades, microbiome study methods have advanced significantly, employing a range of techniques to analyze microbial communities in various environments. Culture techniques, metagenomics, metabolomics, and different imaging techniques are all useful in deciphering *H. pylori*’s intricate relation with the human organism. These modern approaches allow an extensive investigation of the complete microbial population, yielding several insights into its structure and function. For example, metagenomics frequently uses amplicon sequencing of the 16S rRNA gene as a phylogenetic marker or shotgun sequencing, which captures the complete breadth of DNA within a sample, in order to investigate the genetic content of the microbiome, whereas metabolomics quantifies the metabolites generated by the microbial population [58,59,60,61].

In the context of studying *H. pylori* and its abundance using microbiota/microbiome approaches, the variable regions of the 16S ribosomal RNA (rRNA) gene are critical for taxonomic differentiation among bacterial species. The 16S rRNA gene consists of both conserved and variable regions, with the variable regions (V1–V9) providing diversity that can be used to distinguish between different bacteria. For *H. pylori* specifically, the following variable regions of the 16S rRNA gene are particularly useful: The V3 Region is often targeted in many sequencing studies and has shown sufficient variability to discriminate between closely related bacterial species, including *H. pylori.* It provides a good resolution and coverage when analyzing microbial communities. The V4 Region is widely used in microbiome studies, offering good coverage of diverse bacterial taxa, and is often a preferred choice for high-throughput sequencing methods. Its length and sequence variability enable robust identification of *H. pylori* within complex ecosystems. Finally, the V6 and V7 Regions can also contribute to the profiling of *H. pylori* in various microbiota studies. They have shown sufficient variability for distinguishing *H. pylori* and can complement the information obtained from the V3 or V4 Region [14,61]. Combining data from multiple variable regions can enhance the overall coverage and accuracy of *H. pylori* detection and quantification in microbiome studies, helping researchers better understand its role and abundance in different microbiota contexts.

### 3.2. Beyond the Stomach: H. pylori Throughout the Digestive Tract

However, as the use of modern technologies evolved, it revealed that *H. pylori* infection does not only concern the stomach. Its area of influence exceeds the gastric barriers. According to *H. pylori* whole-genome sequencing, this bacterium exhibits extraordinary genetic flexibility and a high rate of gene recombination, which allows continuous adaptation to a changing environment [14].

Although the exact nature of *H. pylori*’s relation with the human microbiome remains elusive, its presence is associated with the alteration of bacterial communities’ composition not only in the gastric microenvironment but also in all digestive tract levels [54,62]. There is proof that *H. pylori* colonizes the oral cavity and may play a role in dental plaque formation and contribute to periodontal disease. It colonizes the stomach lining and can lead to inflammation, increased gastric acid production, and ulceration. *H. pylori* can also be found in the duodenum (the first part of the small intestine). Its presence is linked to duodenal ulcers, which can occur in conjunction with gastric ulcers. The bacterium may disrupt the balance of protective factors and exacerbate duodenal mucosal damage. While the presence of *H. pylori* in the colon is less common, it has been detected there. Its effects in the colon are not as well characterized, but some studies have suggested potential associations with colorectal diseases, warranting further investigation [54].

### 3.3. H. pylori-Induced Dysbiosis and Microbial Imbalance

However, without appropriate management, *H. pylori* can make its way to adult life by creating the premises for several diseases of the digestive tract [45]. Inflammation caused by *H. pylori* impairs the function of wall cells, changes the local pH, and creates an environment that is either favorable to the colonization of other microbes or inhibits their growth [63]. *H. pylori*-induced inflammation can create an environment that favors the growth of specific bacteria as Gram-negative bacteria: inflammatory conditions caused by *H. pylori* can increase the abundance of opportunistic pathogens within the Enterobacteriaceae family, such as *Escherichia coli*, and *Klebsiella* spp. can thrive in an inflamed gastric environment, contributing to potential secondary infections [64]. Certain anaerobic bacteria, such as *Fusobacterium*, can proliferate due to changes in local oxygen concentration and nutrient availability resulting from *H. pylori* infection [65]. Increased levels of Bacteroides may also occur in the presence of inflammation. As for specific phyla, the relative abundance of *Proteobacteria* (which includes *H. pylori* itself and other pathogenic bacteria) can increase during *H. pylori* infection due to inflammatory immune responses [66].

Conversely, *H. pylori* infection can inhibit the growth of certain beneficial bacteria, contributing to dysbiosis in the gut. Beneficial *Lactobacilli*, which play a crucial role in maintaining gut health, may become less prevalent in the presence of *H. pylori*-induced inflammation. Like *Lactobacilli*, *Bifidobacterium* spp. can also be suppressed by *H. pylori*, leading to reduced production of short-chain fatty acids and other protective compounds [50]. *Faecalibacterium prausnitzii*, a butyrate-producing bacterium, which has anti-inflammatory properties, may also decline in abundance due to *H. pylori* infection [67]. Overall, the ratio of *Firmicutes* to *Bacteroidetes* may be disrupted by *H. pylori* infection, as *Firmicutes* (including beneficial bacteria) may decrease while others thrive [66].

## 4. *Helicobacter pylori* and the Oral Microbiome

*Firmicutes*, *Bacteroidetes*, *Proteobacteria*, *Fusobacteria*, and *Actinobacteria* represent the major bacterial phyla of the oral microbiota, while *Fusobacterium*, *Neisseria*, *Haemophilus*, *Porphyromonas*, *Gemella*, *Streptococcus*, *Prevotella*, *Actinomyces*, *Veillonella*, *Alloprevotella*, *Treponema*, *Pseudomonas*, and *Solobacterium* are common at the genus level [58,68]. With more than 700 different types of bacteria, the oral cavity contains the second most varied and diverse microbial community of the body, after the gut [39]. Despite their impressive number, microorganisms can maintain a precise equilibrium under normal physiological settings, but an imbalance in crosstalk will lead to the emergence and progression of illnesses [69,70,71].

As in a bidirectional relationship, microbial shifts are related not only to the factors already cited above but also to the existence of several disorders and to the presence of different pathogens. Found at lower levels in the mouth than in the stomach, *H. pylori* is a good example of interspecies microbial interaction, shaping the composition of the microbiome [72].

### 4.1. Presence and Survival of H. pylori Inside the Oral Cavity

*H. pylori* uses the oral–oral, the fecal–oral, and the gastro–oral routes for dissemination throughout human population [73]. With the oral cavity being the initial entering point for *H. pylori* and its main extragastric reservoir, there is an impressive body of research focused on the relationship between the oral cavity and *H. pylori* infection in adults [74,75]. In children, however, existing data concerning these aspects is scarce.

In our attempt to unravel the intricate interplay between *H. pylori* and oral microbiome, we are addressing some basic questions related to *H. pylori*’s presence in the oral cavity. To begin with, it would be useful to understand what favors its survival at this level, since the mouth offers an environment greatly different from the stomach. Using different methods, *H. pylori* has been found in different niches of the oral cavity, including saliva [76], tongue coating [77], dental plaque [78], as well as dental pulp [79,80,81].

Available information suggests the existence of a mutual relation between *H. pylori* and the oral environment. First, there is direct interaction with the oral cavity’s cells, favored by *H. pylori*’s adhesion and invading abilities [82]. Furthermore, inside the oral cavity, *H. pylori* is protected by a dental biofilm matrix from several hostile factors. With several studies reporting the same facts, dental biofilm offers the ideal pH, temperature, and microaerophilic environment needed for *H. pylori* to survive [75,83]. It is a well-known fact that the biofilm plays several roles in promoting antimicrobial resistance. To begin with, we must admit that the extracellular matrix can act as a physical barrier, preventing antimicrobial agents from penetrating deeply into the biofilm. This barrier can reduce the effective concentration of antibiotics at the bacterial cell surface. Biofilms can also shield bacteria from the host’s immune responses. The structure can impede the recognition and clearance of bacteria by immune cells, allowing persistent infections and making them difficult to treat. Furthermore, within a biofilm, the microenvironment can vary, leading to gradients in pH, oxygen, and nutrient levels. These conditions can impact the metabolic state of the bacteria, allowing some to enter a dormant state that is less susceptible to antibiotics. Bacteria within biofilms can also have increased rates of gene transfer, including plasmids that confer resistance to antibiotics. The proximity of bacterial cells within biofilms facilitates horizontal gene transfer, promoting the spread of resistance genes among different bacterial species [68,72].

The oral cavity can be considered a primary site for *H. pylori* oral colonization as well as for gastric reinfection and there are several studies to consolidate this hypothesis. Bicak [81], Burgers [82], Suzuki [83], and their colleagues reported *H. pylori* to be found in saliva and dental biofilm of children with negative gastric mucosa samples, suggesting that oral *H. pylori* colonization might exist irrespective of *H. pylori* gastric colonization [81,82,83].

### 4.2. Impact of H. pylori on Oral Health and Oral Microbiome Composition

In pediatric patients, along with periodontal health, there was shown to be a favorable correlation between *H. pylori* and dental hygiene [84,85,86]. According to a recent study by Sruthi et al., *H. pylori* may also be found on deep carious surfaces in children’s oral cavities. It has been shown that *H. pylori* is more likely to be found in dental plaques than in saliva specimens, probably because salivary flux may decrease the bacterial levels [87].

Oral health is at risk due to *H. pylori*’s ability to survive in the oral cavity. Numerous scientific reports concluded that the adult population carrying oral *H. pylori* was associated with a higher risk of periodontitis, dental decay, aphthous stomatitis, glossitis, and halitosis compared to their *H. pylori*-negative peers [81,88]. This might be a result of this bacterium’s capacity to alter oral cavity microbiota homeostasis and because of its complex interactions with other members of the oral cavity. Subgingival plaque samples positive for *H. pylori* were associated with more periodontal pathogens than the negative specimens [89]. *P. gingivalis* and *Fusobacterium nucleatum*, well-known pathogens associated with periodontal disease, were reported to be in a positive relation with *H. pylori* [90,91]. *Streptococcus mutans*, the main cariogenic microorganism, together with *H. pylori*, were found to form a symbiotic biofilm, providing *H. pylori* an adequate, microaerophilic environment where bacteria could survive [92]. Furthermore, *H. pylori* seems to have a particular connection with fungi such as *Candida albicans* (*C. albicans*). The bacterium can aggregate with *C. albicans* to form a biofilm that can enhance its survival, or it can be internalized by the fungus and preserve its activity and mobility despite inadequate external milieu, including dryness, high temperature, or even antibiotics [93,94]. As a matter of fact, Chen and colleagues, in their study, discuss the co-existence of *H. pylori* and *C. albicans* in the oral cavity, suggesting a potential role of *Candida* in modulating *H. pylori*’s virulence and persistence [95].

But despite all these beneficial interactions, there are some oral bacterial strains with an inhibitory effect on *H. pylori*’s growth. Some *Prevotella* species, *Streptococcus sobrinus* 6715, *S. mutans* JP2, and *Ingbritt*, displayed a negative impact on *H. pylori* in in vitro conditions [96].

Since the oral host–microbial interplay consists of a bidirectional connection, we must not minimize the influence of *H. pylori* on oral bacterial communities and their interactions, as it can modify their composition and diversity [97,98,99]. In healthy individuals, without any oral and gastric-associated diseases, salivary samples displayed no difference in terms of alpha and beta diversity between *H. pylori*-positive and -negative status. In oral mucosa samples, however, alpha and beta diversity showed different microbial patterns, depending on their *H. pylori* status [76]. The *Haemophilus* genus, *Prevotella gingivalis*, *Prevotella oris*, *Prevotella intermedia,* and *Propionibacterium acnes* all showed decreased oral levels in *H. pylori*-positive patients, known with gastrointestinal disorders, while the *Treponema* genus increased its amounts [100]. The presence of *H. pylori* virulence factors can also make a difference when it comes to microbiome composition. When analyzing CagA-positive *H. pylori*-infected individuals and their tongue plaque samples, Zhao and colleagues found increased abundance of *Proteobacteria* and *Actinobacteria*, while *Firmicutes*, *Bacteroidetes*, and *Fusobacteria* reduced their proportion. In contrast, CagA-negative *H. pylori*-infected individuals presented totally reversed changes, highlighting the influence of virulence factors’ expression on microbiota composition [74].

### 4.3. Effects of H. pylori Eradication Therapy on Oral Microbiota

Finally, we must clarify that *H. pylori*’s presence not only has the capacity to alter the oral microbiome composition but also cause its eradication as well. Although this result upon gastric infection might be successful, antibiotics are not able to overpass the structure of the dental bacterial biofilm [101]. This facilitates *H. pylori* survival in periodontal environments, despite receiving adequate treatment. When analyzing dental pulp samples before initiating eradication therapy and 2 weeks after, Nomura and colleagues reported that nearly all *H. pylori*-positive samples taken from the same teeth remained positive, despite receiving adequate treatment [77]. In other words, there is a higher risk of reinfection with *H. pylori* despite its successful eradication from the gastric milieu, in patients carrying the bacteria in their dental plaques [76].

**Figure 2:**
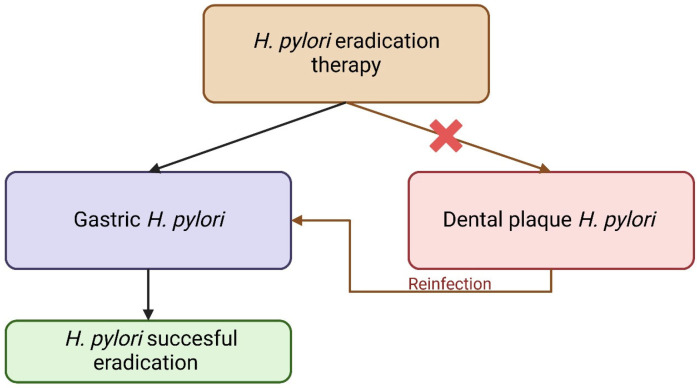
Impact of *H. pylori* eradication therapy on gastric and dental plaque bacteria—hypothetical model.

Figure 2 presents a hypothetical model suggesting that *H. pylori* may persist within dental plaque and serve as a reservoir for gastric reinfection. Although supported by observational and correlative data, this transmission pathway has not been definitively established. Further experimental and longitudinal studies are needed to confirm a causal relationship.

In contrast, oral microbiota, however, holds no protection against eradication therapy. Tawfik and colleagues investigated alterations of the oral–gut microbiome axis of Egyptian teenagers in relation to the eradication therapy of *H. pylori*. Using 16 rRNA sequencing, they analyzed samples of saliva and feces, collected before therapy initiation and 8 weeks after completing the treatment. They reported a reduction in the abundance of several bacterial families of the oral microbiota after finishing eradication therapy, including *Saccharimonadaceae*, *Streptococcaceae*, *Fusobacteriaceae*, *Lachnospiraceae*, and *Staphylococcaceae*, while families like *Prevotellaceae*, *Veillonellaceae*, *Neisseriaceae*, *Carnobacteriaceae*, and *Leptotrichiaceae*, with a predominance of harmful representatives, augmented their levels after antibiotic use. Both fecal and oral samples displayed a reduction in bacterial diversity after receiving eradication treatment [102].

Related to PPI use, although existing data is scarce, there is evidence of a dysbiosis of the oral microbiota associated with their administration. According to one study on healthy adults, a four-week course of esomeprazole administration raised the amount of *Leptotrichia* and *Fusobacterium* in the periodontal pocket, decreased levels of *Neisseria* and *Veillonella* in saliva, and concurrently increased levels of *Streptococcus* in fecal samples. These results suggest that PPIs may affect microbiota in the gut and in the mouth [103]. In contrast with this negative aspect, the eradication therapy can successfully lower the harmful bacteria in the mouth that are enhanced by an *H. pylori* infection, supporting the prevention and management of oral health issues [103].

## 5. *Helicobacter pylori* and the Gastric Microbiome

The gastric environment provides a harsh environment, a result of the extremely low pH, the proteolytic activity of gastric juice, the reflux of bile acids in the stomach, and the antibacterial qualities of nitric oxide, produced from salivary nitrate [104]. Despite these less friendly living surroundings, there is proof that the stomach hosts more than 266 genera, with *Streptococcus*, *Lactobacillus*, *Clostridium*, *Veillonella*, *Haemophilus*, *Enterococcys*, *Pseudomonas*, and *Neisseria* as dominant representatives in healthy adult individuals [105,106].

### 5.1. Gastric Microbiota Alterations in Children with H. pylori Infection

When present, *H. pylori* dominates the gastric microbiota, both in adults and children, with detrimental effects on the variety and abundance of the stomach’s associated microbiome [40,107,108,109]. Available studies regarding the *H. pylori* influence on the gastric microbiota of children are summarized in Table 1.

After analyzing gastric mucosal samples of 51 children who underwent gastric endoscopy to investigate the presence of dyspeptic symptoms (33 negative and 18 positive samples for *H. pylori*), the authors concluded that *H. pylori*’s presence is associated with a decreased bacterial richness and diversity, with the *Helicobacter* genus dominating the gastric bacterial community. *H. pylori*-negative patients display a higher bacterial richness and diversity with an increased relative abundance of *Betaproteobacteria*, *Gammaproteobacteria*, *Bacteroidia*, and *Clostridia classes*. Furthermore, the lowest alpha diversity (*p* = 0.035) was determined in patients *H. pylori*-positive with active superficial gastritis [110]. Miao et al. also found the *Helicobacter* genus (95.43%) to be dominating the gastric microbiota of children with *H. pylori* infection. However, the researchers found no difference in the structure of *H. pylori*-positive children’s microbiota depending on whether they did or did not have a peptic ulcer, suggesting that the main determinant of gastric dysbiosis in these patients might be *H. pylori*’s presence, rather than the stage of the associated disease [38]. With different results, Troncoso et al. reported different compositional patterns of the bacterial communities of the adult gastric mucosa in the presence of *H. pylori*, depending on the gastric epithelium layer’s state, pointing toward a potential influence of the microbiota on adult gastric disease initiation and progression [109].

### 5.2. Immune Response and Gastritis Development in Pediatric H. pylori Infection

Similar results were reported by Zheng et al., after evaluating the gastric and duodenal mucosa of 122 children who went through an upper digestive tract endoscopy for gastrointestinal complaints. *H. pylori* infection is linked to a reduction in gastric bacterial variety and richness, with the *Helicobacter* genus monopolizing the composition of gastric microbiota. Furthermore, it favors gastritis development as it is associated with an altered immune response. There is proof that *H. pylori*-positive patients with a secondary gastric dysbiosis display greater levels of TGF-b1, FOXP3, IL-10, and IL-17A, all of which were associated with enhanced CD4+ T-cell and macrophage cells [111]. In addition to these microbiota-related changes, another study of Zheng et al. focused on children with duodenal ulcers demonstrates that *H. pylori* colonization diminishes a potentially genotoxic microbial population of the stomach mucosal layer of patients presenting duodenal ulcers [112,114]. The fact that bacterial communities with specific nitrate reductase functions are reduced in *H. pylori*’s presence in children contradicts the theory derived from adult research, sustaining *H. pylori*’s carcinogenic profile. On the other hand, it might as well be proof of the specific features of *H. pylori* infection, in adult and pediatric populations [113].

Another study, incorporating both children and adults, concluded that *H. pylori*-positive children with gastrointestinal complaints have a different gastric microbiota composition compared to their adult peers, with lower *Firmicutes phylum* representative taxa and more non-*Helicobacter* *Proteobacteria*. Furthermore, only children with positive Helicobacter displayed an increased expression of proinflammatory markers in their gastric mucosa, with high levels of TGFb and IL-10 in addition to expanded FOXP3 transcripts. In contrast to pediatric report results, *H. pylori*-negative and -positive adults present high similarities with regards to their gastric microbiota composition [113,115].

### 5.3. Impact of H. pylori Eradication Therapy on Gastric Microbiota

Eradication therapy for *H. pylori*, which often includes a combination of antibiotics and proton pump inhibitors, can have differing impacts on gastric microbiota between children and adults. In children, the gastric microbiota is still developing, leaving it potentially more susceptible to alterations than in adults. Studies suggest that eradication of *H. pylori* in children can lead to a marked change in the diversity and composition of gastric microbiota [54].

Related to IPP use, beside oral dysbiosis, IPPs were associated with gastric dysbiosis as well. Secondary to their administration, their use interferes with normal gastric functions and favors a rise in bacterial load levels as well as in bacterial translocation [116,117]. In their research study, Paroni Sterbini and colleagues reported that *Streptococcaceae* levels could serve as an independent marker of gastric microbiome-associated dysbiosis, secondary to IPP use [118]. Furthermore, hypochlorhydria and a raised pH value result in a microbial shift towards bacteria with nitrate/nitrite reductase functions simultaneously with a reduction in microbial communities’ diversity [119,120]. Oral bacteria’s growth, including *Pepto-streptococcus stomatis*, *Parvimonas micra*, *Streptococcus anginosus*, *Dialister pneumosintes*, and *Slackia exigua*, could also be encouraged by the increase in gastric pH level. Information of great importance on these bacteria, eliciting distinct metabolic processes, could be involved in gastric cancer pathogenesis [120]. *H. pylori* is known to activate inflammatory signaling pathways, particularly through the production of virulence factors like CagA. This can result in an increased expression of proinflammatory cytokines (e.g., IL-6, IL-8) and contribute to a chronic inflammatory state within the gastric mucosa. A study by Ou et al. reported that *H. pylori* infection facilitates cell migration through the activation of the NF-kB signaling pathway, which is crucial for inflammation and has implications for tumor progression in gastric cancer [121].

### 5.4. Long-Term Microbiota Recovery and Health Outcomes

As for gastric microbiota recovery after antibiotic eradication treatment, most available studies regarding the eradication treatment effect on gastric microbiota are focused on adult patients. Most authors share the idea that following the eradication of *H. pylori*, the gastric microbiota can exhibit both transient and long-term effects, depending on multiple factors such as individual host characteristics, age, the presence of other gastrointestinal conditions, and the specific eradication therapies used. In children, gastric microbiota is still developing, making it potentially more susceptible to alterations than in adults [2,7].

Many individuals may experience transient alterations in microbiota soon after eradication, often associated with a state of dysbiosis. Studies indicate that within a short period post-eradication, there may be significant and rapid changes in the composition of the gastric microbiota. This early phase can be characterized by a shift towards increased microbial diversity. However, for some, particularly in the pediatric population, these changes can lead to beneficial long-term outcomes characterized by sustained microbial diversity. The distinction between transient and long-term recovery highlights the complexity of gastric microbiota dynamics and their associations with health [66].

Miao and colleagues observed that within one month after *H. pylori* eradication, patients exhibited pronounced changes in gastric microbiota composition, reflecting a transient state before potential stabilization toward a baseline level [38]. The recovery phase may also include a transient state of dysbiosis. Research by Kakiuchi et al. reported that some individuals experienced gastrointestinal symptoms attributed to dysbiosis following eradication, lasting for several months before restoring to an equilibrium [122].

Conversely, other studies demonstrate that while initial changes are transient, the eradication of *H. pylori* can lead to long-term beneficial shifts in gastric microbiota composition. For example, a longitudinal study by He et al. showed that over an extended period post-eradication, gastric microbiota in patients exhibited sustained increases in potentially beneficial bacteria [123]. In children, Zhou et al. found that following successful eradication, participants maintained an enhanced gut microbial diversity for up to a year, suggesting a longer-term benefit not typically seen in adults [124].

Longer-term effects vary by population. A cohort study by Mao et al. demonstrated that adults who were *H. pylori*-negative retained altered microbial profiles which were linked to the development of gastric and systemic diseases over multiple years, indicating that while some individuals may return to a pre-eradication state, others may experience persistent alterations impacting health [125].

In addition to its influence on gastric microbiota richness and diversity, it seems that *H. pylori* might have an important influence on its functions, as well. *H. pylori*-positive patients display an increase in cancer and infectious diseases, while -negative patients are associated with increased levels of carbohydrate, amino acids, and lipid metabolism [72]. The relationship between *H. pylori* infection and gastric cancer is well documented. Chronic inflammation and changes in microbial function can contribute to carcinogenesis. The study of Ou and colleagues highlights how *H. pylori* facilitates cellular processes that may impact disease progression and prognosis in gastric cancer, while Yang and colleagues reported different gastric microbiota compositions in the adult population within observational groups, associated with high or low gastric cancer risk [114,126].

In adults, while there are changes in microbiota following eradication, these changes may be less pronounced due to a more stable and established microbial community in the adult stomach. Research highlights that eradication can reduce certain bacterial populations while increasing others, resulting in a heterogeneous response to therapy [127].

In children, increasing microbial diversity post-eradication may have additional positive implications, potentially reducing the risk of subsequent gastrointestinal diseases. A study by Chen et al. indicated that children with successful eradication exhibited lower rates of functional dyspepsia and improved overall gastrointestinal health. Conversely, in adults, changes in gastric microbiota following *H. pylori* eradication might be associated with the development of post-eradication dysbiosis, leading to symptoms like gastritis or other functional gastrointestinal disorders [128].

## 6. *Helicobacter pylori* and the Intestinal Microbiome

With the purpose of investigating the impact of *H. pylori* over the gut microbiota bacterial composition, in terms of current infection and post-eradication therapy in children, we will discuss the available reports focused on this matter, as summarized in Table 2.

### 6.1. Intrafamilial Transmission of H. pylori and Shared Gut Microbiota Patterns

There is proof that there is an intrafamilial transmission of *H. pylori*, but Osaki et al. questioned whether siblings of *H. pylori*-positive infants share the composition of their intestinal microbiota. When examining 18 fecal samples of *H. pylori*-positive infants and their family members using 16 rRNA sequencing, the authors reported no discernible changes between *H. pylori*-negative and *H. pylori*-positive children and adults regarding the *Firmicutes*/*Bacteroidetes* ratio. However, the low patient numbers in each group may be the cause of this uniform result [129].

### 6.2. Gut Microbiota Changes in Symptomatic vs. Asymptomatic Children

The alterations in intestinal microbiota due to *H. pylori* infections differ considerably between symptomatic and asymptomatic children as well, with symptomatic cases typically showing reduced diversity and a predominance of harmful bacteria. Benavides-Ward and colleagues examined the intestinal microbiota of asymptomatic *H. pylori*-positive children and compared the results with the intestinal microbiota of healthy controls. Children who tested positive for *H. pylori* were twice as likely to have an increased richness and diversity of bacterial communities in their colon, including *Clostridium* spp. from *Firmicutes phylum* and *Prevotella* spp., among *Proteobacteria phylum*’s representatives [130]. An augmented diversity of bacterial communities in the gut of *H. pylori*-positive patients was also reported in adults, with a different microbiota composition when compared to *H. pylori*-negative individuals [134].

Furthermore, the research of El Amrousy et al. found that children with symptomatic infections exhibited increased levels of pathogenic bacteria like *Clostridium difficile* and *Salmonella* spp., while *Lactobacillus* spp., *Bacteroides fragilis*, *Methanobrevibacter smithii*, and *Escherichia coli* decreased in their levels in *H. pylori*-positive children. Conversely, the microbiota of asymptomatic children tended to maintain higher levels of protective microbes [50]. The authors reported similar results in adults, with *Lactobacillus* spp. levels being influenced by *H. pylori*’s presence [135].

In a similar approach, Lapidot et al. found no relation with respect to gut bacterial communities’ richness and the presence of *H. pylori* in asymptomatic children. All of this aside, *H. pylori* is able to alter the composition of the gut microbiome, with an overrepresentation of *Prevotella copri* and *Eubacterium biforme* in healthy *H. pylori*-positive children, while *Clostridium*, *Ruminococcus,* and *Coprococcus* spp. are associated wih decreased levels [131].

Symptomatic infections often correlate with elevated inflammatory markers within the gut. A study by Michaelkiewicz and colleagues suggested that children with symptomatic *H. pylori* infections had higher levels of cytokines and inflammatory mediators, which can further alter the gut microbiota composition, promoting dysbiosis [136].

Symptoms such as abdominal pain, nausea, and vomiting in symptomatic children may be associated with specific shifts in gut microbiota [136]. For example, Yang et al. pointed out that a symptomatic child exhibited a distinct microbial signature, including changes that could contribute to functional gastrointestinal disorders [66].

### 6.3. Impact of H. pylori Eradication Therapy on Gut Microbiota

As for the outcomes of the *H. pylori* eradication therapy on the intestinal microbiota composition, several studies came to the same conclusion; despite the immediate dysbiosis associated with the eradication treatment, after a period of 2 to 3 months, the gut microbiota was restored to its pretreatment levels [122,132,136]. In children, a significant pediatric study by Gotoda et al. compared the microbiota of *H. pylori*-positive children before and after eradication, reporting an increase in beneficial bacteria like *Lactobacillus* and *Bifidobacterium* post-therapy, which are often linked with improved gut health [132]. A recent systematic review of 24 papers focused on *H. pylori* eradication’s influence on adult gut microbiota found that while most studies reported a significant decrease in the gut microbiota’s alpha diversity shortly after eradication, no additional changes were seen for more than six months. Short-term dysbiosis of the gut microbiota caused by bismuth quadruple treatment is characterized by an excess of *Proteobacteria* and a reduction in *Bacteroidetes* and *Actinobacteria*. Adverse effects during eradication therapy may be attributed in part to an increase in gut *Proteobacteria* [137]. As already discussed in gastric microbiota, eradication treatment not only induces compositional changes in the gut microbiota but also significantly alters its functionality, impacting various metabolic and inflammatory pathways [138]. *H. pylori* influences the functionality of the gut microbiota by altering metabolic pathways, particularly those related to the fermentation of polysaccharides and the production of short-chain fatty acids (SCFAs). Studies show that *H. pylori* infection can lead to a decrease in microbial diversity, thus impacting the metabolic capacity of the gut microbiota to produce SCFAs, which are essential for maintaining gut health and modulating immune responses [139]. However, a study by Hu et al. demonstrated that dual therapy recommended for *H. pylori* eradication has minimal negative effects on gut microbiota composition and SCFA production such as butyrate, which are pivotal in maintaining epithelial integrity and regulating inflammation in the gut [140].

**Figure 3:**
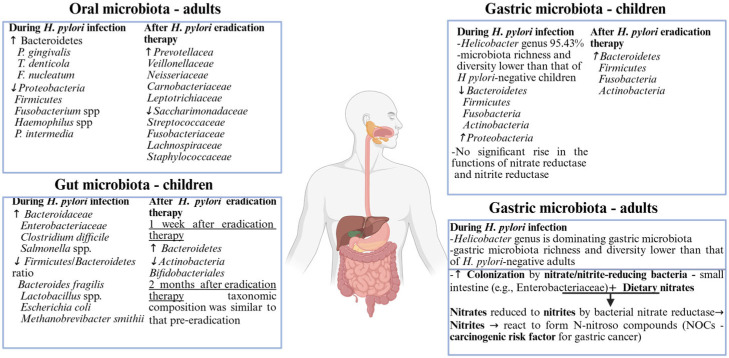
Digestive tract microbiota compositional pattern based on *H. pylori* infection presence/eradication therapy. Abbreviations: ↑, increased level; ↓, decreased level.

With regards to the available pediatric research, all three studies [112,124,140] evaluated adolescents who received vanoprazol, amoxicillin, and claritromicin except one group of patients from Kakiuchi et al.’s study. In addition to the triple therapy scheme, Kakiuchi et al. associated biofermin-R, a probiotic strain of *Enterococcus faecium* 129 BIO 3B-R. It seems that probiotic supplementation increased bacterial species richness during the eradication treatment and prevented stool consistency modification, in comparison to triple therapy alone [140]. Figure 3 presents a comparative summary of microbiota changes associated with *H. pylori* infection and eradication across different anatomical sites and age groups (adults vs. children). It highlights observed shifts in microbial composition during and after *H. pylori* infection in the oral, gastric, and gut microbiota.

## 7. Geographic Limitations of Pediatric *H. pylori* Microbiome Research

Most of the pediatric studies investigating the impact of *H. pylori* on the digestive microbiome originate from Eastern Europe and East Asia. This regional concentration of research likely reflects factors such as higher *H. pylori* prevalence in these areas, the availability of specialized gastroenterology centers, and established research networks focusing on infectious gastrointestinal diseases. However, this geographic bias limits the generalizability of findings. Data from Africa, Latin America, and other underrepresented regions are scarce, despite these areas often having a high *H. pylori* burden and distinct environmental, dietary, genetic, and healthcare contexts that could shape microbiome composition and host–microbe interactions differently [53,54,141]. The lack of representative global data hampers our ability to fully understand regional variations in microbiota responses to *H. pylori* infection and eradication therapy in children, and it highlights the need for multi-center, cross-continental pediatric microbiome studies.

## 8. Conclusions

Colonizing the digestive tract of more than half of the people worldwide, *H. pylori* has been the subject of numerous research papers. Associated with the development of several digestive and extradigestive disorders, it represents a source of endless controversy. To treat or not to treat *H. pylori* infection is a question that has prevailed over time. With both beneficial and harmful effects on one’s state of health, the presence of *H. pylori* in the digestive tract has always been a reason of concern for physicians, especially for those treating the pediatric population. As technology has evolved, it has given us the means to unravel the multiple facets of *H. pylori* infection and shed light on the intricate connection of the bacterium with the human organism.

Due to its unique interaction with the human body, the human microbiome plays an essential role in defining and enhancing the host’s physiology. Found in all digestive tract levels, *H. pylori*’s presence seems to define the microbial compositional patterns, with specific changes in children and adults. *H. pylori* infection in the pediatric population will always be a matter of interest due to its potential to determine long-term complications and significant morbidity. Understanding the fact that the dysbiosis caused by *H. pylori* is not limited to the gastric microenvironment, but affects the entire gastrointestinal tract, will provide a new perspective on *H. pylori* infection and hopefully will offer more effective therapeutic options. With positive results in adult studies, microbiota manipulation might offer valuable results in the management of pediatric *H. pylori* infection as well, but future research is required.

## Figures and Tables

**Table 1:** Studies assessing the influence of *H. pylori* infection on children’s gastric microbiota.

Study	Subjects/Methods	Study Type, Samples, and Techniques	Gastric Microbiota Before Eradication Therapy	Gastric Microbiota After Eradication Therapy	Other Findings	Study Limitations
Miao et al., 2019 [38]	-37 symptomatic children *H. pylori*-positive (23 nonpeptic ulcer and 14 peptic ulcer) -18 symptomatic children *H. pylori*-negative	-cross-sectional, observational study -16S rRNA amplification -gastric mucosa	*H. pylori*-positive -*Helicobacter* genus 95.43% -microbiota richness and diversity lower than that of *H. pylori*-negative children ↓ *Bacteroidetes* *Firmicutes* *Fusobacteria* *Actinobacteria* ↑ *Proteobacteria* -no difference in microbiota structure between *H. pylori*-positive children with or without peptic ulcer *H. pylori*-negative ↑ *Bacteroidetes* *Firmicutes* *Fusobacteria* *Actinobacteria*	*H. pylori*-positive 4 weeks after treatment lower community richness -similar community diversity compared to *H. pylori*-negative *↑Bacteroidetes* *Firmicutes* *Fusobacteria* *Actinobacteria*	-the pathways of infection diseases and cancer were of higher abundance in *H. pylori*-positive group -the pathways of metabolic diseases were lower than *H. pylori*-negative group -the abundance in the pathways of amino acid metabolism, lipid metabolism, and carbohydrate metabolism was lower in *H. pylori*-positive group than *H. pylori*-negative group	-small cohort, which may limit statistical power and generalizability -a cross-sectional design cannot establish causality between *H. pylori* infection and microbiota alterations -single-center study results may not be representative of broader pediatric populations or different geographic/ethnic groups
Llorca et al., 2017 [110]	51 children with dyspeptic symptoms (18 *H. pylori*-positive, 33 *H. pylori*-negative)	-cross-sectional, observational study -16S rRNA bacterial gene sequencing -gastric mucosa	*H. pylori*-positive children vs. -negative children lower bacterial richness and diversity ↑ *Proteobacteria* phylum *Helicobacter* genus ↓ *Firmicutes*		-children with *H. pylori* infection associated with a lower body mass index -*Serratia*—1st *Pseudomonas*—2nd *Staphylococcus*—3rd most abundant genus in all patients	-small cohort, which may limit statistical power and generalizability -a cross-sectional design cannot establish causality between *H. pylori* infection and microbiota alterations -single-center study results may not be representative of broader pediatric populations or different geographic/ethnic groups
Zheng et al., 2021 [111]	122 children with GI symptoms (57 *H. pylori*-positive, 65 *H. pylori*-negative)	-cross-sectional, observational study -16S rRNA bacterial gene sequencing -gastric mucosa	*H. pylori*-positive ↑ *Helicobacter* taxa *H. pylori*-negative ↑ *Achromobacter* *Devosia* *Halomonas* *Mycobacterium* *Pseudomonas* *Serratia* *Sphingopyxis* *Stenotrophomonas*		*H. pylori*-positive children vs. -negative children ↑ expression of FOXP3, IL-10, TGF-b1, and IL-17A ↑ CD4+T cell and macrophages	-relatively small cohort, which may limit statistical power and generalizability -a cross-sectional design cannot establish causality between *H. pylori* infection and microbiota alterations -single-center study results may not be representative of broader pediatric populations or different geographic/ethnic groups -immune analysis limited to mucosal markers—systemic immune responses were not assessed, which may underestimate host–microbe interactions
Zheng et al., 2022 [112]	23 children with duodenal ulcer (15 *H. pylori*-positive, 8 *H. pylori*-negative)	-cross-sectional, observational study -16S rRNA bacterial gene sequencing -gastric mucosa	*H. pylori*-positive children vs. -negative children ↓ bacterial richness and diversity of gastric microbiota ↑ *Helicobacter* taxa		*H. pylori*-positive children vs. -negative children ↓ pathways of carbohydrate metabolism, amino acid metabolism, lipid metabolism, and signal transduction	-small cohort, which may limit statistical power and generalizability -a cross-sectional design cannot determine causality between *H. pylori* infection, microbiota shifts, and duodenal ulcer pathology -niche population (only duodenal ulcer patients)—findings may not generalize to asymptomatic or non-ulcer pediatric populations -single-center study—regional/ethnic specificity reduces generalizability -lack of longitudinal data—no follow-up to assess whether microbiota changes persist after *H. pylori* eradication or ulcer healing
Brawner et al., 2017 [113]	26 symptomatic patients *H. pylori*-positive (12 children; 14 adults) 60 symptomatic patients *H. pylori*-negative (33 children; 27 adults)	-cross-sectional, observational study -16S rRNA bacterial gene sequencing -gastric fluid	*H. pylori*-positive children vs. -negative children ↓ *Actinobacteria* class ↓ *Streptococcaceae* *Actinomycetales* *Moraxellaceae* *Carneobacteriaceae* family ↑ *Helicobacter* genus *H. pylori*-positive adults vs. -negative adults N *Actinobacteria* class ↑ *Helicobacter* genus		-children with *H. pylori* infection were associated with reorganized stomach microbiota at several taxonomic levels -*H. pylori*-positive children vs. -positive adults: ↓ *Firmicutes* ↑ non-*Helicobacter* *Proteobacteria* -*H. pylori*-positive adults have a similar composition of the gastric bacterial communities to that of -negative adults -*H. pylori*-positive children, when compared with non-infected children and infected adults, expressed significantly more FOXP3 transcripts and increased TGFb expression and levels of IL10 in their gastric mucosa	-small cohort, limiting statistical power to detect subtle microbial shifts -single geographic region (Chile)—findings may not generalize to other populations with different diets, genetics, or *H. pylori* strain distributions -potential confounders—limited control for prior antibiotic exposure, dietary factors, or socioeconomic influences that may affect microbiota

Abbreviation: 16S ribosomal RNA, 16S rRNA; *Helicobacter pylori*, *H. pylori*; gastrointestinal, GI; ↑, increased level; ↓, decreased level.

**Table 2:** Studies assessing the influence of *H. pylori* infection on children’s gut microbiota.

Study	Subjects/Methods	Study Type, Samples, and Techniques	Gut Microbiota Before Eradication Therapy	Gut Microbiota After Eradication Therapy	Other Findings	Study Limitations
Yang et al., 2019 [66]	50 children with *H. pylori*-induced gastritis 42 children with *H. pylori*-negative gastritis 62 healthy controls	-cross-sectional, observational study -16S rRNA bacterial gene sequencing -fecal sample	*H. pylori*-positive gastritis ↑ *Bacteroidaceae* *Enterobacteriaceae* ↓ *Firmicutes*/*Bacteroidetes* ratio *H. pylori*-negative gastritis ↑ *Bacteroidaceae* *Enterobacteriaceae* ↓ *Firmicutes*/*Bacteroidetes* ratio Healthy controls ↑ *Lachnospiraceae* *Bifidobacteriaceae* *Lactobacillaceae*		-compared to healthy controls, the fecal microbiome of children with gastritis alone and gastritis related to *H. pylori* was different, indicating that gastric inflammation may influence the gut microbiota	-cross-sectional design—cannot establish temporal or causal links between *H. pylori* infection, gastritis, and gut microbiota dysbiosis -niche cohort—only children with gastritis were included; findings may not apply to healthy or asymptomatic children -sample size—relatively limited, reducing power to detect subtle differences -stool samples only—gut microbiota may not fully reflect gastric mucosal microbiota, limiting direct relevance to gastric pathophysiology
Amrousy, D.E. et al., 2023 [50]	50 *H. pylori*-positive adolescents 50 healthy controls	-cross-sectional, observational study -RT-PCR -fecal sample	*H. pylori*-positive children vs. controls ↑ *Clostridium difficile* *Salmonella* spp. ↓ *Bacteroides fragilis* *Lactobacillus* spp. *Escherichia coli* *Methanobrevibacter smithii*		-predictive of *H. pylori* infection is an increased abundance of *Salmonella* spp. and *Bifidobacterium* spp., a greater prevalence of *C. difficile*, and a decreased abundance of *Lactobacillus* spp.	-cross-sectional design—does not allow inference about causality or temporal relationships between *H. pylori* infection and gut microbiota changes-sample size—relatively small, limiting statistical power and ability to detect subtle microbial differences-single-country, single-center cohort (Egypt)—findings may not generalize to other adolescent populations with different diets, environments, or *H. pylori* strains-stool-based microbiota analysis only—gut microbiota may not fully reflect gastric or duodenal microbiota relevant to *H. pylori* pathogenesis
Osaki et al., 2018 [129]	5 *H. pylori*-positive children and the members of their families (4 *H. pylori*-positive mothers, 3 *H. pylori*-positive fathers, 1 *H. pylori*-negative mother, 2 *H. pylori*-negative fathers, 4 *H. pylori*-negative siblings)	-cross-sectional, observational study -16S rRNA sequencing -18 fecal samples	*H. pylori-*positive children ↑ *Parasutterella* *H. pylori-*negative group ↑ *Erysipelotrichaceae* family *Clostridiaceae* *Ruminococcaceae* *H. pylori-*negative parents ↑ *Ruminococcus* *Faecalibacterium*		-despite the limited sample size, results suggested that members of the same family had comparable gut bacterial community composition	-small sample size -lack of longitudinal data -the study design cannot conclusively determine whether similarity in microbiota facilitates transmission, or whether *H. pylori* infection itself alters the microbiota, leading to the observed similarities
Kakiuchi et al., 2021 [122]	16 *H. pylori*-positive adolescents	-prospective, longitudinal, multicenter observational study -16S rRNA gene/DNA/amplicon sequencing -fecal sample		1–2 days after eradication therapy alpha-diversity was lost immediately after eradication therapy ↓Actinobacteria 3 months after eradication therapy α-diversity recovered to pretreatment levels	-adolescents receiving vonoprazan fumarate-containing triple therapy for *H. pylori* eradication experienced an immediate dysbiosis following treatment; however, three months later, the gut microbiota restored to pretreatment levels -regarding unfavorable outcomes, triple treatment was determined to be safe for adolescents	-small sample size -no control group—the study lacked a placebo or healthy non-infected adolescent comparator group, which limits contextual interpretation of the microbiota changes -no antibiotic susceptibility data—the study did not conduct *H. pylori* antibiotic resistance or treatment susceptibility testing, which could influence eradication outcomes
Zhou et al., 2021 [124]	16 *H. pylori*-positive children receiving standard triple therapy (TT) 15 *H. pylori*-positive children receiving sequential therapy (ST) 16 *H. pylori*-positive children receiving bismuth-based quadruple therapy (BT) 15 *H. pylori*-positive children receiving concomitant therapy (CT)	-prospective, comparative cohort study -16S rRNA sequencing -fecal samples		2 weeks after eradication marked decline in the alpha diversity in all groups ↑ *Proteobacteria* in ST, BT, CT ↑ *Escherichia* *Shigella* *Klebsiella* *Enterococcus* *Streptococcus* in all groups 1 year after eradication relative abundance of all phyla in all groups did not differ from those at baseline	2 weeks after eradication ↓ SCFA-producing bacteria, such as *Bacteroides*, *Faecalibacterium*, and *Phascolarctobacterium* -gut microbiota was less perturbed by TT and ST -all 4 current therapies lead to transient dysbiosis of the gut microbiota, but these changes returned to almost the pre-eradication level 1 year post-eradication	-comparative, non-randomized allocation—participants were assigned to treatment arms per physician’s decision—not randomized—raising potential selection bias -no healthy (non-infected) or untreated control group was included to contextualize microbiota changes attributable to *H. pylori* infection versus antibiotic impact
Benavides-Ward et al., 2018 [130]	28 asymptomatic children *H. pylori*-positive 28 healthy children	-cross-sectional observational study -16S rDNA sequencing -fecal samples	*H. pylori*-positive children vs. -negative children ↑ *Proteobacteria*, *Clostridium* *Firmicutes* *Prevotella* ↓ *Bacteroides*		-*H. pylori*-positive children had twice as many chances of having an elevated variety and number of bacterial communities in their colon -growth stunting was more frequent in *H. pylori*-infected children compared to non-infected peers -despite being clinically asymptomatic, infected children still showed microbiota disruption and nutritional impact, highlighting subclinical effects of *H. pylori* infection	-small sample size -cross-sectional observational study cannot determine causality or temporal relationships between *H. pylori* infection and microbiota changes -conducted in a specific population (Peruvian children), possibly limiting ability to generalize findings to broader or diverse populations -potential influences such as diet, environmental factors, antibiotic exposure, socioeconomic status, or other health variables were not controlled for or adjusted
Lapidot et al., 2021 [131]	93 asymptomatic children *H. pylori*-positive 70 healthy children	-cross-sectional observational study -16S rRNA gene sequencing -fecal sample	*H. pylori*-positive children vs. controls ↑ *Prevotella copri* *Eubacterium biforme* *Coriobacteriaceae* ↓ *Clostridium* spp. *Coprococcus* spp. *Ruminococcus* spp.		-intestinal microbiome diversity, including bacterial abundance and richness, did not significantly correlate with *H. pylori* infection	-potential socioeconomic confounding as the authors acknowledge that socioeconomic status (SES) is both strongly linked to *H. pylori* infection and gut microbiome composition, complicating the interpretation of any direct effects -though controls were included, controlling fully for lifestyle differences (beyond measured SES) remains a challenge in observational cross-sectional assessments
Gotoda et al., 2018 [132]	8 teenagers *H. pylori*-positive	-prospective, longitudinal, observational study -16S rDNA sequencing -fecal samples		1 week after eradication therapy ↑ *Bacteroidetes* ↓ *Actinobacteria* *Bifidobacteriales* 2 months after eradication therapy taxonomic composition was similar to that pre-eradication	-one week after starting treatment, alpha diversity decreased, and two months later, it nearly returned to pre-treatment levels -the number of bacterial species at different times did not significantly change in terms of beta diversity	-small sample size -short follow-up period as longer-term recovery beyond that remains unexplored -absence of an untreated or uninfected control group makes it difficult to distinguish therapy effects from normal microbiome fluctuations
Kakiuchi et al., 2020 [133]	BFR− group 31 *H. pylori*-positive adolescents receiving VPZ-containing triple therapy BFR+ group 35 *H. pylori*-positive adolescents receiving VPZ-containing triple therapy + Biofermin-R (BFR-*Enterococcus faecium* 129 BIO 3B-R)	-randomized controlled trial -16S rDNA sequencing -fecal samples		BFR− group ↓ alpha diversity *Collinsella* spp. *Bifidobacterium* spp. BFR+ group ↑ *Blautia* microbial species richness -less drastic shifts in beta-diversity and retained more stable microbial community structures *↓* incidence of diarrhea	-during *H. pylori* eradication therapy, biofermin-R in combination with VPZ-based therapy resulted in decreased stool softness and increased microbial α-strain diversity	-small sample size -short follow-up window—microbiota was assessed only before treatment and immediately after the 7-day therapy. No long-term follow-up samples were collected to evaluate whether observed changes persisted or normalized over time -clinical outcome scope: while diarrhea incidence was tracked, other outcomes—like *H. pylori* eradication rate or long-term gut health—are not described in available summaries

Abbreviations: 16S ribosomal RNA, 16S rRNA; 16S ribosomal DNA, 16S rDNA; reverse transcription polymerase chain reaction, RT-PCR; *Helicobacter pylori*, *H. pylori*; standard triple therapy, TT; sequential therapy, ST; bismuth-based quadruple therapy, BT; concomitant therapy, CT; short-chain fatty acid, SCFA; vanzoprazol, VPZ; Biofermin-R, BFR; ↑, increased level; ↓, decreased level.

## Data Availability

Not applicable.
